# Unlocking the Role of Exercise on CD4+ T Cell Plasticity

**DOI:** 10.3389/fimmu.2021.729366

**Published:** 2021-10-25

**Authors:** Chloé D. Goldsmith, Thomasina Donovan, Nicole Vlahovich, David B. Pyne

**Affiliations:** ^1^ Research Institute for Sport and Exercise, University of Canberra, Canberra, ACT, Australia; ^2^ Australian Centre for Health Services Innovation and Centre for Healthcare Transformation, School of Public Health and Social Work, Faculty of Health, Queensland University of Technology, Brisbane, QLD, Australia; ^3^ Faculty of Health, University of Canberra, Canberra, ACT, Australia; ^4^ School of Psychology and Life Sciences, Faculty of Science, Engineering and Social Sciences, Canterbury Christ Church University, Canterbury, United Kingdom

**Keywords:** immune, epigenetics, metabolism, DNA methylation, chromatin remodeling, histone modification, mitochondria

## Abstract

A hallmark of T cell ageing is a loss of effector plasticity. Exercise delays T cell ageing, yet the mechanisms driving the effects of exercise on T cell biology are not well elucidated. T cell plasticity is closely linked with metabolism, and consequently sensitive to metabolic changes induced by exercise. Mitochondrial function is essential for providing the intermediate metabolites necessary to generate and modify epigenetic marks in the nucleus, thus metabolic activity and epigenetic mechanisms are intertwined. In this perspective we propose a role for exercise in CD4+ T cell plasticity, exploring links between exercise, metabolism and epigenetic reprogramming.

## Introduction

Exercise can improve the efficacy of certain cancer therapies targeting kidney (1), bladder, testicular, and head and neck cancers (2). Moreover, exercise alone can improve the outcomes for individuals with diseases including cardiovascular (3), kidney disease (4, 5) and diabetes (6). It appears that the mechanism(s) underlying the benefits of exercise on disease outcomes is related to augmentation of cytokine profiles, however, there is little known about the involvement of immune cells in this process.

**Figure 1:**
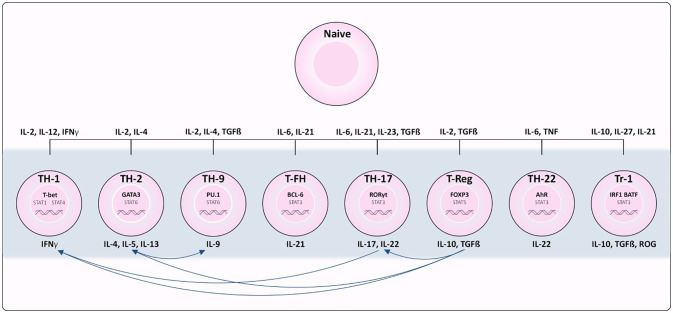
Differentiation of CD4+ T cell subsets from naïve T cells. Cytokines required for polarization as well as the transcription factors and cytokines produced by each subset are indicated. Th1 cells depend on STAT1 activation and expression of transcription factor (TF) *TBX21*. Th2 depends on IL-4 activation of STAT6 resulting in up-regulation of TF GATA3. Th17 differentiation is associated with IL-6/IL-21 and STAT3 induced expression of RORγT. TGFβ and IL22 are required for activating TF FOXP3 in Regulatory T cells (Treg) differentiation. Tfh differentiation is dependent on STAT3, IL6 and IL21 to induce BCL6 expression. Th9 polarization relies on IL4, TGFβ and STAT6 activation to induce expression of PU.1 and IRF4. In Tr1 cells, IL-10, IL-27, and IL-21 promote STAT3 activation, and IL-27 promotes expression of TF’s IRF1 and BATF. Lastly, STAT3 stimulated by IL-6 and TNF induce expression of AHR in Th22 cells.

The establishment and maintenance of immune responses, homeostasis and memory depends on T lymphocytes (T cells). T cells originate from multipotent hematopoietic progenitors that migrate to the thymus for maturation, selection, and subsequent export to the periphery. The events in the thymus determine lineage commitment (CD4/CD8+ lineages), as well as the fate of mature T cells. Commitment to the CD8+ lineage results in cells with specialized cytotoxic potential, while commitment to the CD4+ lineage (**Figure 1**) results in naïve cells with broader differentiation potential of T helper (Th) and regulatory T cell (Treg) subsets. Despite previous *in vitro* studies emphasizing terminal commitment in T cells, it has become clear that plasticity is widespread in the CD4+ T cell lineage and potentially integral for maintaining host immunity (7).

Recent evidence has revealed that CD4+ T cell subset stability can be dependent on coordination between metabolic and epigenetic mechanisms [reviewed in (8)]. Considering the profound influence exercise has on metabolism, the role of exercise on CD4+ T cell stability is poorly characterized. Furthermore, there is little known about the interplay between exercise, metabolism, epigenetics and the stability/plasticity of CD4+ T cells. In this perspective, we highlight recent work exploring the role of exercise on CD4+ T cells and examine links between metabolism and epigenetic reprogramming in these immune cell subsets.

## CD4+ T Cell Characterization and Stability

Each CD4+ T cell subset can be characterized by its ability to sense different inductive cytokines, program the expression of distinct transcription factors, and function by producing select cytokines and chemokine receptors to control specific pathogens or prevent immune pathology [reviewed in (9) and summarized in **Figure 1**]. Th1 differentiation is dependent on STAT1 activation and expression of transcription factor (TF) *Tbx21* (10). Conversely, IL-4 signals activate STAT6 resulting in up-regulation of TF *Gata3* and Th2 polarization (11). Th17 differentiation is associated with IL-6/IL-21 and STAT3 induced expression of the TF RORγT (12), and TGFβ is required to signal expression of TF FOXP3 for regulatory T cell (Treg) differentiation. T follicular helper (T-fh) differentiation is dependent on STAT3, IL-6 and IL-21 to induce BCL6 as the major TF. Presence of IL-4 and TGFβ stimulate STAT6 pushing the differentiation of Th9 cells, expressing TF’s PU.1 and IRF4. STAT3 stimulated by IL-6 and TNF are needed for differentiation of Th22 cells expressing AhR as their major TF. In T regularity type 1 (Tr1) differentiation, IL-10, IL-27, and IL-21 promote STAT3 activation, and IL-27 promotes pioneering TFs IRF1 and BATF (13), however there are many TFs that can then be activated in Tr1s depending on a variety of factors (14). While lineage commitment to CD4+ T cell subsets at one time appeared to be stable, emerging evidence has revealed the capacity of polarized T cells to change their phenotype, and repolarize towards mixed or alternative fates (15).

## CD4+ T Cells Exhibit Plasticity

Plasticity of CD4+ T cells can be defined as the ability of a single cell to take on characteristics of many T cell subsets simultaneously, or at different times, during the course of its life cycle. With age, T cells acquire a terminally differentiated stage, losing plasticity and compromising the capacity of the immune system to respond to new antigenic challenges (16). Understanding the factors that induce/contribute to plasticity are imperative to determine the mechanism of T cell ageing. Early studies were able to induce CD4+ T cell re-polarization using cytokine cocktails *in vitro* towards almost any fate (Reviewed in (15)). However, not all effects have been replicated *in vivo* models. Those identified, are summarized in **Figure 1**. Briefly, Th17 cells are considered highly plastic, with fate mapping studies indicating they can give rise to Th1 cells (17) as well as intermediate phenotypic cells implicated in disease (18). Th1 and Th2 cells have displayed interconversion (19). Treg cells have displayed plasticity towards Th17 cells, but also can co-express effector cytokines from other Th lineages (20). While this research has been highly valuable to improving our understanding of the environmental conditions inducing T cell plasticity, recent advances in single-cell RNA sequencing has revealed a great deal of heterogeneity among populations of what were perceived to be homogeneous T cell subsets based on cell surface markers. Intra-cell heterogeneity was recently demonstrated with single Th17 cells that exhibited a range of phenotypes from pathogenic to regulatory in nature (21). Therefore, there is a need to better characterize the identity of immune cell subsets in order to have a clear picture of the directionality of plasticity. Nevertheless, these studies are fueling the hypothesis that CD4+ T cells can exhibit phenotypic plasticity in response to changing contexts.

## Exercise Modulates the Number and Activity of CD4+ T Cells

The effect of exercise on immune health has been widely studied in various population cohorts, however, exercise-research targeting CD4+ T cells is less represented. Nevertheless, exercise has been shown to modulate both the number and activity of CD4+ T cells, with an increased number of CD4+ T cells observed in the blood of athletes after training (22). Furthermore, physical fitness can modulate the concentration of immune cell subsets (VO_2_max exhibiting a large correlation (*r* = 0.69) with Treg populations in the blood outside of training). Moreover, exercise can alter the balance of Th17/Tregs (increased Th17 and decreased Treg populations) improving chronic heart failure outcomes in a murine model (23). In athletes post training, increased Th1, Th17 and Treg populations have been observed. In contrast, no changes in Th2 cell concentrations have been identified immediately after exercise or after a recovery period. Interestingly, only Th17 cell populations remained elevated into the recovery period (24). To our knowledge, there are yet to be any reports of Th9, Th22 or Tr1 cell modulation by exercise in healthy subjects. Furthermore, exercise and sport-related studies rarely include comprehensive immune-analysis, tending to instead focus resources on determining the concentration of plasma-cytokines, leaving the immune cells involved unexplored. In reports that have identified CD4+ T cell subsets, the mechanisms driving these observations are not characterized, however, it is likely that metabolism plays an integral part.

## Metabolic Drivers of CD4+ T Cell Plasticity

Metabolic programs engaged by T cells directly affect their identity and function. Following T-cell Receptor (TCR) stimulation, CD4+ T cells rapidly acquire metabolites required for cellular processes, and even the method of Adenosine triphosphate (ATP) generation can vary depending on immune cell identity. CD28 signaling is important for Treg activation, triggering upregulation of GLUT1 expression controlling the metabolic switch to glycolysis, and increasing cellular glucose uptake (25). Notably, once activated Tregs do not use glycolysis, and instead rely on fatty acid oxidation to feed the Tricarboxylic acid cycle (TCA) cycle and generate energy through oxidative phosphorylation (26).

The TF hypoxia-inducible factor 1α (HIF1α) is positively regulated by PI3K–AKT–mTOR signals. HIF1α induces the expression of genes required for glycolysis when stabilized by low oxygen availability. Treg cells prevent the induction of HIF1α expression and glycolysis during TCR stimulation by dampening the PI3K– AKT–mTOR pathway (27). In addition to promoting glycolysis, HIF1α has an important role in Th17 cell polarization, by directly inducing the expression of RORγt and supporting its function. The transcriptional activity of HIF1α is also opposed by the transcriptional repressor B cell lymphoma 6 (BCL-6), which competes for binding to many of the same genes, preventing the induction of glycolytic genes that may be detrimental to the T-fh cell program (28).

Glutamine metabolism generates large amounts of the metabolite α-ketoglutarate (αKG) and has been linked to Th1-Treg cell plasticity. αKG is required for Th1 cells but blocks Treg differentiation in an mTORC1- dependent manner. In a similar way, regulation of fatty acid metabolism and downstream cholesterol biosynthesis by CD5 antigen-like (CD5L) in Th17 cells can act as a crucial checkpoint promoting regulatory *versus* pathogenic activities within the Th17 cell subset (29).

Metabolism changes markedly across the lifetime in a sex-specific manner. Significant alterations in lipid, amino acid and energy metabolite profiles have been observed in cohorts of ageing men and women, with menopause also implicated (30). These metabolic changes can have consequences for CD4+ T cell identity, including downregulation of glycolytic metabolism potentially leading to the loss of plasticity observed in ageing. While the crucial role of metabolic programming in T cell physiology is emerging, many of the potential impacts of ageing on metabolic homoeostasis for T cell plasticity remain unexplored.

These studies highlight that fluctuations in nutrients, oxygen levels and energy sources present in the environment can influence CD4+ T cell plasticity and influence pathogenicity. Understanding changes in metabolic pathways in CD4+ T cells is part of the key to unraveling mechanisms of plasticity and immunosenescence. However, further research is required to disentangle the interplay between metabolic pathways, and the gatekeeper of cellular states, epigenetics.

## Epigenetic Drivers of CD4+ T Cell Plasticity

Epigenetics refers to the heritable layer of information on top of a genomic sequence that regulates gene expression. Generally, this information encompasses DNA methylation, histone modifications and noncoding RNAs. In mammalian genomes, DNA methylation commonly includes the addition of methyl groups to cytosines (5mC) and adenines (6mA) (31). While cytosines can also be hydroxy-methylated (5hmC) (32), formylated (5fC) and carboxylated (5caC), their role in gene regulation has not been well studied. Importantly, in certain tissues mitochondrial DNA can also be methylated, however the function of these marks is not well elucidated (33). Histone modifications include acetylation, methylation, phosphorylation and ubiquitination of histone tails. Histone modifications linked to open chromatin are classified as permissive (H3K4me3, H4ac), and those linked to closed chromatin are considered repressive (H3K27me3). Moreover, histone modifications can indicate the activity of gene enhancers (poised: H3K4me1 and/or active: H3K27ac). DNA methylation and histone modifications regulate gene expression by governing chromatin accessibility, and recruit important TFs and epigenetic writers, readers, and erasers.

Epigenetic marks govern the plasticity of T cells by lowering or raising the threshold between cellular states (34). Whole genome mapping of permissive and repressive histone modifications in different CD4+ T cell lineages can reveal the presence of a mixed ‘poised’ state in the promoter of lineage‐specific TFs. For example, in Th17 cells, the *FOXP3* promoter is not epigenetically repressed, potentially allowing Th17 to Treg plasticity. Moreover, *TBX21* was decorated with permissive marks in Th2 lineage, allowing plasticity between Th1 and Th2 (19).

The plasticity of immune cells has implications for disease. In intestinal inflammation models Th17 cells can be highly plastic, developing a ‘Th1 like’ phenotype by expressing IFNG STAT4 and TBET, driving disease development (18). The phenotype of these pathogenic Th1 ex Th17 cells is associated with an increase in DNA methylation in *IL17A* locus, and a decrease in DNA methylation in *TBX21* and *IFNG* (35). These data support the hypothesis that environmental cues can induce immune cell plasticity. However, the upstream factors responsible for driving epigenetic re-programming in T cells are not well understood.

## Metabolites and Cofactors Required for Epigenetic Modifications

**Figure 2:**
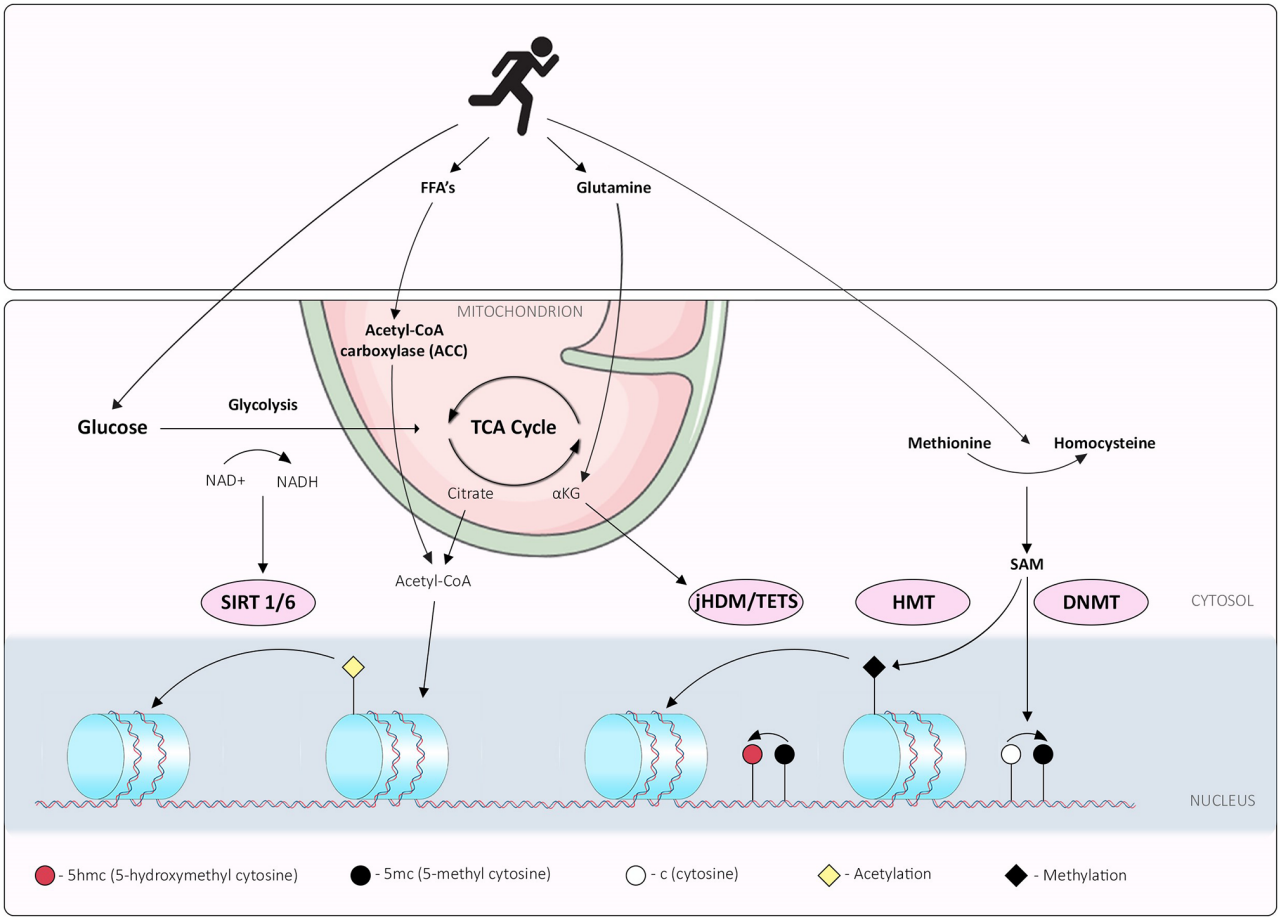
Interplay between exercise, metabolism and epigenetic mechanisms; overview of exercise induced metabolic pathways that synthesize metabolites or cofactors required for epigenetic marks. Exercise promotes glycolysis, which determines the NAD+/NADH ratio, integral for the activities of sirtuin histone deacetylases. Free Fatty Acid (FFA) and glutamine metabolism can be perturbed by exercise, potentially affecting acetyl CoA derived from either FFA or the TCA cycle and maintenance of histone acetylation. The histone demethylation reaction, catalyzed by Lysine-specific histone demethylase 1A (LSD1), involves the reduction of co-factor flavin adenine dinucleotide (FAD) to FADH2, and release of formaldehyde as a by-product. Histone demethylases family Jumonji-C domain contain histone demethylases (jHDM), catalyze a distinct demethylation reaction from LSD1. This reaction utilizes α-ketoglutarate (αKG), oxygen and Fe (II) as co-factors, and releases succinate and formaldehyde as by-products; this mechanism is also used by TET family enzymes that hydroxylate the 5-methylcytosine of DNA. Exercise limits glutamine metabolism reducing available αKG. DNA methylation reactions are affected through one carbon metabolism; S-adenosylmethionine (SAM) is produced via one-carbon metabolism from methionine to homocysteine by the enzyme methionine adenosyltransferase; exercise increases peripheral homocysteine affecting the methionine: homocysteine cellular ratios. DNA methyl-transferases (DNMTs) and histone methyl-transferases (HMTs) transfer methyl groups to DNA and histones *via* the same mechanism, utilizing a methyl group from SAM to generate methylated DNA/histones and a molecule of S-adenosyl homocysteine (SAH).

Several compounds formed through different stages of metabolism have been recognized as playing a part in different epigenetic mechanisms (summarized in **Figure 2**). Briefly, flux through glycolysis determines the NAD^+^/NADH ratio which is important for the activities of sirtuin histone deacetylases, while acetyl CoA derived from the TCA cycle is important for maintaining histone acetylation (36). The histone demethylation reaction, catalyzed by Lysine-specific histone demethylase 1A (LSD1), involves the reduction of co-factor flavin adenine dinucleotide (FAD) to FADH_2_, and the release of formaldehyde as a by-product. As recycling of FAD requires converting molecular oxygen to hydrogen peroxide, cellular redox status might influence the availability of FAD and thus the activity of LSD1. Subsequently a family of histone demethylases named Jumonji-C domain contain histone demethylases, catalyze a distinct demethylation reaction from LSD1 (37). This reaction utilizes αKG, oxygen and Fe (II) as co-factors, and releases succinate and formaldehyde as by-products. This mechanism is also used by TET family enzymes that hydroxylate the 5-methylcytosine of DNA. Conversely, DNA methylation reactions are affected through one carbon metabolism; S-adenosylmethionine (SAM) is produced *via* one-carbon metabolism from methionine by the enzyme methionine adenosyltransferase (38). DNA methyl-transferases (DNMTs) and histone methyl-transferases (HMTs) transfer methyl groups to DNA and histones *via* the same mechanism, utilizing a methyl group from SAM to generate methylated DNA/histones and a molecule of S-adenosyl homocysteine (SAH). While this is not an exhaustive list, these metabolites are imperative for the maintenance of epigenetic marks and thus, cellular identity. Considering the potential of metabolic flux to be induced by environmental cues, including exercise, exploration of the roles of metabolite availability on cellular plasticity is of high importance.

## Exercise Affects Metabolic Programs Important for Epigenetic Mechanisms in CD4+ T Cells

Substrate utilization during exercise will vary depending on the exercise type, intensity, and duration. During low to moderate intensity exercise, the main substrates are glucose, glutamine and fatty acids; with glucose becoming a more prominent fuel source as intensity increases (39). Considering the importance of mitochondria in cell metabolism, the effects of exercise in mitochondria have been studied extensively. Although it is expected that exercise-related adaptations mainly affect muscle mitochondria, exercise can influence surrounding cells *via* the availability of metabolites. Here we link studies highlighting the effects of exercise on metabolic programs, and the availability of metabolites, required for epigenetic remodeling events (**Figure 2**).

Glutamine is an important fuel for Th1 cells. Skeletal muscle is the major tissue involved in glutamine production and known to release glutamine into the bloodstream at a high rate. Skeletal muscle plays a vital role in maintenance of the key process of glutamine utilization in immune cells, and therefore, the activity of skeletal muscle may directly influence the immune system. According to the glutamine hypothesis, a decrease in plasma glutamine concentrations, brought about by heavy exercise limits the availability of glutamine for cells of the immune system that require glutamine for energy and nucleotide biosynthesis. Thus, factors that directly or indirectly influence glutamine synthesis or release could theoretically influence the function of CD4+ T cells (40, 41).

Acetyl-CoA carboxylase (ACC) is produced in various metabolic pathways and serves as an acetyl group donor of histone acetyltransferase, which is the critical step of acetylation (**Figure 2**). Activated Th1 cells express lactate dehydrogenase A (LDHA) to support aerobic glycolysis, allowing Th1s to maintain high ACC levels, which in turn, promote histone acetylation at important loci including *IFNG*, and thus expression of inflammatory cytokines. Importantly, in the absence of LDHA, mice were protected from autoimmune diseases associated with an abundance of IFNγ (42). Interestingly, ACC is decreased in human skeletal muscle during exercise (43). Considering the importance of ACC for maintenance of histone acetylation in T cells, and the link with immune pathologies, it is plausible that exercise-associated reductions in ACC could affect the maintenance of histone acetylation. Collectively this sequence of events could induce changes in expression of vital identity genes such as *IFNG* in Th1 cells. Such environmental cues could be key for inducing plasticity of CD4+ T cells.

Homocysteine is an amino acid, whose conversion from methionine is an integral step for one-carbon metabolism (**Figure 2**). Elevated plasma homocysteine levels are linked with diseases including cardiovascular disease and cancer. In the immune system, homocysteine is known as a potent concentration-dependent T cell activator promoting differentiation as well as potentiating activation-induced cell death and apoptosis (44). More specifically, homocysteine is linked with a Th17 and Tfh cell imbalance in individuals with abdominal aortic aneurysm (45). Interestingly, homocysteine levels can be manipulated by exercise. In rats, homocysteine plasma levels are affected by exercise in a dose-dependent manner (46), accompanied by decreased liver SAM/S-adenosylhomocysteine. In humans, plasma homocysteine is affected inversely by exercise type, with aerobic exercise increasing plasma levels and resistance training decreasing levels (47). Considering the link between homocysteine and CD4+ T cell stability, and exercise-induced effects on homocysteine levels, it is conceivable that exercise-specific homocysteine manipulation can modulate DNA methylation landscapes of CD4+ T cell subsets. These examples provide further evidence for the potential mechanisms of exercise-induced T cell plasticity.

As discussed earlier, regulation of Free Fatty Acid (FFA) metabolism in Th17 cells is a crucial checkpoint in promoting regulatory *versus* pathogenic phenotypes (29). The lipid profile and saturation level [Polyunsaturated/Saturated Fatty Acids (PUFAS/SFAs)] of available FFAs was involved in regulation of Th17 pathogenicity, with SFAs linked to pathogenic profiles and PUFAs with non-pathogenic. The modulation of FFAs by exercise has been well studied; notably, exercise increases the plasma unsaturated/saturated fatty acid ratio (48). The link between FFAs and epigenetics has been well studied (49), however, this research has focused on dietary consumption of fats and dietary intervention studies rather than the effect of exercise. Despite this, we can extrapolate that similar epigenetic consequences would be observed with exercise interventions that affect lipid profiles and FFA availability. However, there is a need for targeted research in this realm to decipher conclusively the role of exercise on FFAs and epigenetic regulation of T cells.

It is important to note that there are other pathways that could facilitate exercise-induced CD4+ T cell plasticity, such as, contraction-induced modulators of gene expression in skeletal muscle, muscle hypertrophy and mitochondrial biogenesis. However, here we have focused on the direct consequences of exercise on metabolic pathways which could affect the maintenance of epigenetic marks in peripheral CD4+ T cells. This metabolic-epigenetic axis is central to interrogating more closely potential mechanisms of exercise-induced plasticity in these specific immune cell subsets.

## Conclusions

CD4+ T cells include a diverse population with highly varied function. Plasticity is exhibited between CD4+ T cell subsets and linked to numerous maladies such as development and exacerbation of autoimmune disorders and tumorigenesis. Exercise has been shown to effect CD4+ T cells, however, our understanding of the mechanisms surrounding these changes is limited. We propose that exercise could alter CD4+ T cell identity through metabolic responses to exercise, which in turn, affect the availability of metabolites and induce epigenetic remodeling events. To substantiate these claims, more research is needed to profile the epigenetic landscape of CD4+ T cells in response to exercise at the single cell level, identifying intermediate cell subsets, and deciphering the role of exercise on immune cell plasticity.

## Data Availability Statement

The original contributions presented in the study are included in the article/supplementary material. Further inquiries can be directed to the corresponding author.

## Author Contributions

CG drafted the manuscript. CG, TD, NV, and DP reviewed and provided critical feedback on the manuscript. All authors contributed to the article and approved the submitted version.

## Conflict of Interest

The authors declare that the research was conducted in the absence of any commercial or financial relationships that could be construed as a potential conflict of interest.

## Publisher’s Note

All claims expressed in this article are solely those of the authors and do not necessarily represent those of their affiliated organizations, or those of the publisher, the editors and the reviewers. Any product that may be evaluated in this article, or claim that may be made by its manufacturer, is not guaranteed or endorsed by the publisher.

## Abbreviations

T cells, T lymphocytes; 5caC, 5-carboxylcytosine; 5fC, 5-Formlcytosine; 5hmC, 5-Hydroxymethylcytosine; 5mC, 5-Methylcytosine; 6mA, N6-methyldeoxyadenosine; ACC, Acetyl-CoA carboxylase; ATP, Adenosine triphosphate; BCL-6, B cell lymphoma 6; CD5L, CD5 antigen-like; DNMT, DNA methyl-transferases; FAD, flavin adenine dinucleotide; FFA, Free Fatty Acid; HIF1α, hypoxia-inducible factor 1α; HMT, histone methyl-transferases; LDHA, lactate dehydrogenase A; LSD1, Lysine-specific histone demethylase 1A; PUFA, Polyunsaturated Fatty Acids; SAH, S-adenosyl homocysteine; SAM, S-adenosylmethionine; SFA, Saturated Fatty Acids; TCA, Tricarboxylic acid cycle; TCR, T-cell Recptor; TF, Transcription Factor; T-fh, T follicular helper; Th, T helper; Tr1, T regularity type 1; Treg, Regulatoy T cell; αKG, α-ketoglutarate.
